# Editorial

**DOI:** 10.3897/zookeys.1177.111533

**Published:** 2023-08-30

**Authors:** Caroline S. Chaboo, Michael Schmitt

**Affiliations:** 1 Department of Entomology, University of Nebraska State Museum, University of Nebraska-Lincoln, W-436 Nebraska Hall, Lincoln, NE 68583-0514, USA University of Nebraska State Museum, University of Nebraska-Lincoln Lincoln United States of America; 2 Universitaet Greifswald, Allgemeine & Systematische Zoologie, Loitzer Str. 26, D-17489 Greifswald, Germany Universitaet Greifswald Greifswald Germany

This is volume 9 in the series ‘Research on Chrysomelidae (RoC-9)’. This started from RoC-1 in 2008, named as a series to reflect the dream of Pierre Jolivet for a long tradition into the future. This dream was built on previous special volumes of Chrysomelidae research that emerged largely through Jolivet’s efforts since 1988 (‘Biology of Chrysomelidae’) and from various international symposia. For example, ‘Special Topics in Leaf Beetle Biology’, edited by David Furth, emerged from the International Congress of Entomology held in Iguassu, Brazil in 2000.

We hope that Chrysomelidae-focused symposia will continue to be organised in the future and that the RoC series will continue to assemble diverse research and researchers into these valuable volumes that accelerate and enrich research on Chrysomelidae. This family of beetles is one of the most speciose on Earth, is ancient, and their herbivorous nature altogether make it an enormously significant group. These special issues also reflect the cooperation and collaboration within the Chrysomelidae community. Today, we have a valuable partnership with the ZooKeys production team that helps manage the process and prepare the high-quality layouts and publications.

RoC-9 assembles nine articles, four from the 10^th^ International Symposium on the Chrysomelidae (26^th^ International Congress of Entomology in Helsinki, Finland, July 2022) and five from independent submissions. This range of topics, questions, and problems reflects the dynamic and diverse research being conducted today on leaf beetles.

Since RoC-8 was published in 2019, we lost several figures central to recent decades of leaf beetle research: Andrzej Warchałowski (17 September 1927 – 20 September 2019), Dieter Siede (14 November 1955 – 10 August 2020), and Pierre Hippolyte Auguste Jolivet (12 October 1922 – 30 September 2020). We also commemorate Terry Lee Erwin (1 December 1940 – 11 May 2020) whose seminal 1982 paper ‘Tropical forests: Their richness in Coleoptera and other arthropod species’ was based largely on leaf beetle data. We dedicate this volume to the memory of these deceased colleagues, whose scientific legacy forms the foundation of future research and whose tireless zeal remains both an incentive and obligation for us. We are confident that the leaf beetle workers’ community will continue to widen knowledge on Chrysomelidae and will also maintain future ‘Research on Chrysomelidae’ issues.

We thank all authors and reviewers for their share in the production of RoC-9, and also the ZooKeys team at Pensoft Publishers, especially Yordanka Banalieva and Nathalie Yonow. They made our work as editors a joy and a reward.


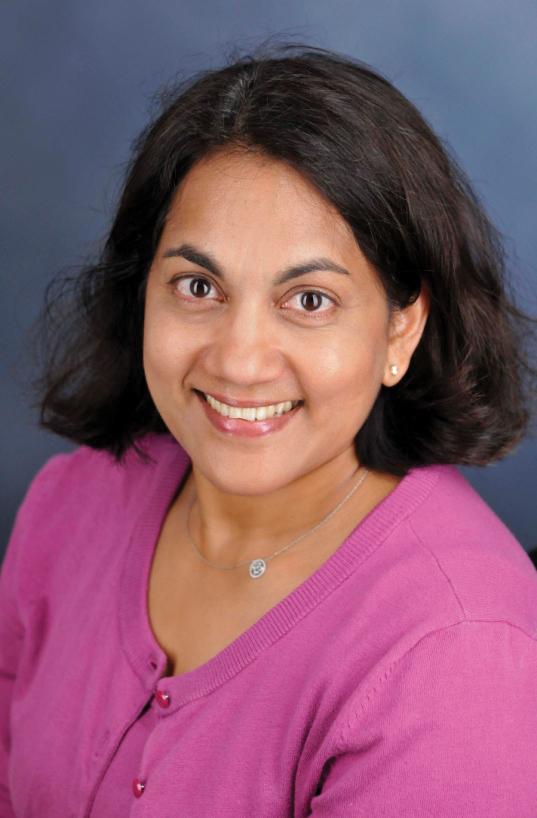


Caroline S. Chaboo


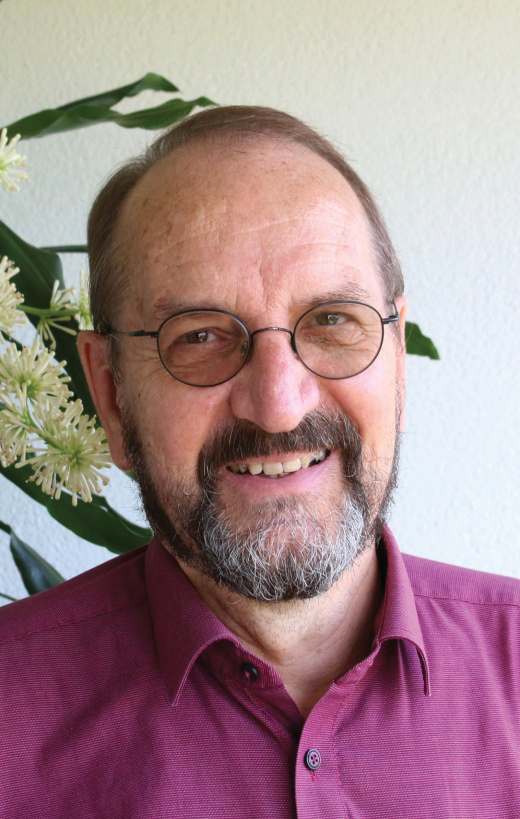


Michael Schmitt

## ﻿Additional information

### Author ORCIDs

Caroline S. Chaboo https://orcid.org/0000-0002-6983-8042

Michael Schmitt https://orcid.org/0000-0001-7377-3643

### Data availability

All of the data that support the findings of this study are available in the main text.

